# Improving Access to Lactation Consultation and Early Breast Milk Use in an Outborn NICU

**DOI:** 10.1097/pq9.0000000000000130

**Published:** 2019-01-04

**Authors:** Kristen T. Leeman, Kimberly Barbas, Julia Strauss, Shannon Adams, Karen Sussman-Karten, Alyssa Kelly, Margaret G. K. Parker, Anne Hansen

**Affiliations:** From the * Division of Newborn Medicine, Department of Pediatrics, Boston Children’s Hospital, Boston, Massachusetts, USA; ^ Harvard Medical School, Boston, Massachusetts, USA; † Department of Nursing, Boston Children’s Hospital, Boston, Massachusetts, USA; ‡ Department of Pediatrics, Boston Medical Center, Boston, Massachusetts, USA; # Boston University School of Medicine, Boston, Massachusetts, USA.

## Abstract

**Introduction:**

Increasing the use of breast milk in critically ill neonates is an important priority to improve neonatal care. Lactation consultants (LCs) educate mothers about evidence-based benefits of breast milk and provide technical support. LC support can lead to increased breastfeeding initiation. The project aim was to improve access to lactation services for mothers of patients admitted at <48 hours after birth to an exclusively outborn level III/IV neonatal intensive care unit (NICU).

**Methods:**

The interventions included (1) implementation of an automatic electronic admission order for a lactation consult, (2) initiation of a daily lactation team notification, (3) assignment of a consistent NICU LC, and (4) targeted education. The percent of mothers who received lactation consults, the time to the first consultation, and the percent of patients receiving breast milk at 7 days of age were measured over 32 months and analyzed using statistical process control charts.

**Results:**

The lactation consultation rate increased significantly from 74% to 88% with a shift in the mean by statistical process control chart analysis that was sustained over time. Concurrently, the time to first lactation consultation significantly decreased from hospital days 5 to 3.3, and variation decreased. Rates of breast milk use at 7 days of age also significantly increased from 75.6% to 89.6%.

**Conclusions:**

Targeted quality improvement interventions led to an increased rate of lactation consultations, decreased time to first lactation consult, and increased rate of breast milk use at 7 days of age. These interventions could feasibly be implemented in similar referral NICU settings.

## Abstract 2

Supplemental Digital Content is available in the text.

## INTRODUCTION

The World Health Organization and the American Academy of Pediatrics recognize the important health benefits of mother’s milk and recommend its use in neonatal populations.^1–7^ Several evidence-based practices improve breast milk use in the neonatal intensive care unit (NICU), including parental education, early and frequent milk expression, and skin to skin care.^8–11^ Breastfeeding support can be provided by bedside nurses, peer counselors, and other trained lactation educators [certified lactation counselors or international board-certified lactation consultants (IBCLC)]. Lactation consultants (LCs) are critical resources to promote breast milk use in vulnerable neonatal populations,^12,13^ including critically ill newborns transferred to referral hospitals for their medical care.^7,12,13^

In the NICU setting, mothers must express breast milk using pumps to establish and maintain a breast milk supply, as most newborns are too ill to tolerate direct feeding at the breast in the first days after birth. Early initiation is a key factor in sustained breast milk production.^8^ Education, motivation, and support for breast milk expression are essential to successful breast milk production.^14^ Lactation consultation with mothers of very preterm infants has been shown to increase initiation and breast milk feeding without increasing maternal stress and anxiety.^15^ Mothers of NICU infants have identified inconsistent advice and lack of assistance as a contributor to lactation failure.^16^ Encouragement of mothers to breastfeed does not lead to maternal feelings of coercion but instead is helpful to make informed decisions about breastfeeding.^17^

The global aim of this project was to increase early breast milk use in an outborn level III/IV NICU. We identified a delay in mothers’ receiving lactation consultations as a modifiable factor that could influence early breast milk expression and use. Thus, this project aimed to improve the percent of mothers of patients admitted at <48 hours of age to a level III/IV NICU who received lactation consultations to >85% over a 32-month period. The team also tracked time to first consultation and breast milk use.

## METHODS

### Setting

The Boston Children’s Hospital (BCH) NICU is an academic tertiary/quaternary referral center caring for outborn newborns with complex medical and surgical conditions. More than 650 infants are admitted each year with approximately 150 to 200 of these infants admitted at <48 hours after birth. All infants are cared for by a multidisciplinary team that may include neonatologists, general surgeons, neonatal and surgical fellows, neonatal nurse practitioners, neonatal nurses, nutritionists, LCs, social workers, pharmacists, and child life specialists.

**Fig. 1.**
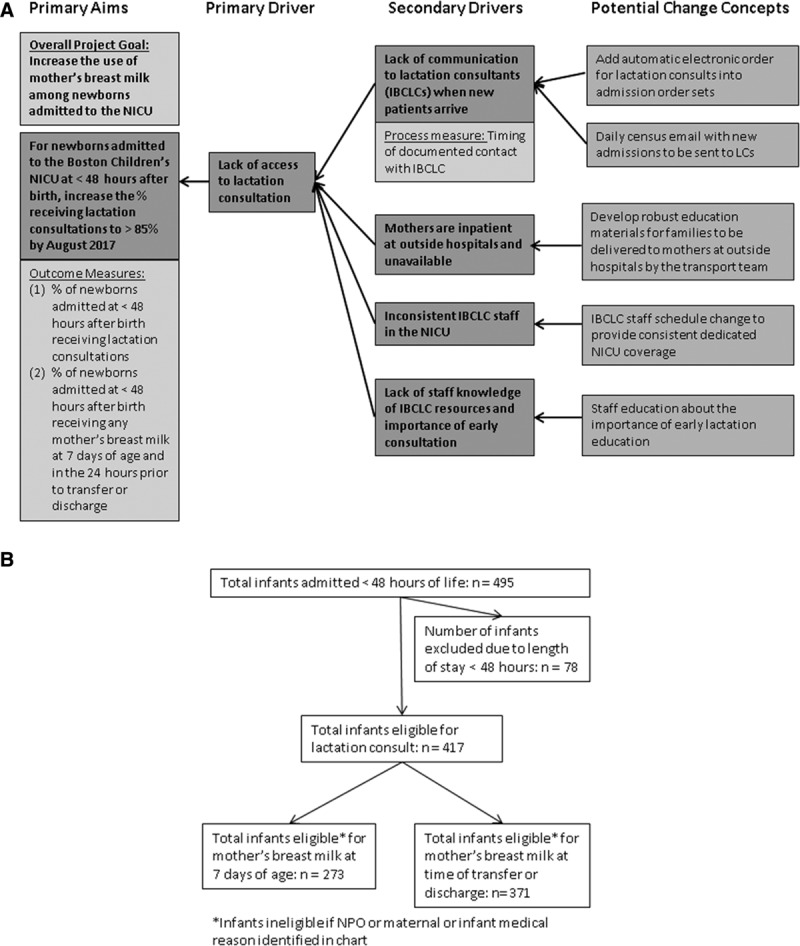
Key driver diagram and flow diagram of infant inclusion criteria. A. The key driver diagram outlines potential change concepts that affect secondary drivers, primary drivers, and primary project aims. B. Graphic of patient eligibility, total infants admitted, and excluded before analysis. For mothers’ breast milk eligibility analysis at 7 days of life and time of transfer or discharge, ineligible infants included patients with an NPO status or chart documentation of a maternal or infant medical reason.

In 2015, BCH NICU joined the Massachusetts statewide neonatal quality improvement collaborative, as 1 of 10 level 3 NICU teams participating in a 3-year project (2015–2017) focused on increasing use of mother’s milk. The BCH team modified the neonatal quality improvement collaborative key driver diagram to focus on a unique set of drivers in the outborn NICU environment (Fig. 1A). One key driver identified was a delay in access to IBCLC support for new mothers. These mothers deliver their critically ill newborns at outside hospitals. The newborns then transfer to the BCH referral center NICU, and the mother is either transferred to a neighboring maternal center or visits after she is discharged from the outside hospital. This unique situation can lead to a delay in lactation initiation, decreased access to pumping equipment, and high levels of stress and anxiety. All of these factors can negatively impact milk supply, the motivation for pumping, and maternal confidence in the ability to express milk, ultimately decreasing the dose and duration of mother’s milk received.

IBCLCs with pediatric nursing experience comprise the Lactation Support Program at BCH. The IBCLCs support mothers of newborns throughout the hospital; however, 0.5 IBCLC full time equivalents (FTEs) are dedicated to the NICU only. Before this study, there was not a specific, consistent IBCLC assigned to the NICU and consults were staffed throughout the hospital in the order they were received.

#### Interventions.

In a quality improvement project targeted toward improving mother’s access to lactation support services, an interdisciplinary team developed a Key Driver Diagram (Fig. 1A) and changed concepts to test in multiple Plan-Do-Study-Act cycles. Prospectively, 4 tests of change were implemented to improve the rate of lactation consultations for eligible infants and decrease time to first lactation consult. These 4 interventions included are as follows:

##### Addition of a New Order for Lactation Consultation Into the Admission Electronic Order Set.

In January 2016, the team added an order for lactation consultation into the NICU physician admission order sets. This order generates an electronic notification to the lactation team. Before this project, the ordering prescriber would have to specifically search for the lactation consult order and add it separately. This change eliminated extra steps in the ordering process and also increased prescriber awareness of the need for a lactation consult for new admissions.

##### Start of a Daily Email Notification to the Lactation Support Team of New NICU Admissions.

In July 2016, an automatic daily email report of new NICU admissions started. This system allows the IBCLC to review all new overnight admissions for eligibility for lactation consult. The IBCLC can then inquire about the potential need for consultation, availability of mother, and plan the ideal timing of their visit. This automated system eliminated the need for NICU staff members to page the covering IBCLC for new consultations.

##### Staffing Change to a Dedicated, Consistent NICU IBCLC.

Before this project, there was not a dedicated IBCLC assigned to the NICU; consults were staffed based on availability at the time of consult request. In October 2016, to create an environment of accountability, ownership, and increased visibility, the lactation program adjusted the staff scheduling model to identify 1 consistent IBCLC assigned to the NICU (0.5 FTE). This constant presence has led to improved communication, education, and relationships between the NICU and IBCLC staff. Staff have expressed that it is helpful to know who to contact for lactation support clearly and that this promotes a culture of unit ownership and dedication from the NICU IBCLC.

#### Education to Staff and Families.

To increase awareness of the project, the team educated staff about the importance of breast milk and lactation consultation services by delivering high-yield bulleted education points, both via email and an electronic board in the break room. To focus on outreach, the transport team also directly provided education to mothers at outside hospitals about early pumping, benefits of breast milk, and lactation resource contact information. The transport team delivered the 1-page educational document to mothers before transporting their infant.

### Data Collection and Measures

The improvement team first performed a 12-month retrospective chart review for all admissions to the NICU to assess the baseline rate of consultation, time to the first lactation consult, and rate of mother’s milk use at the time of transfer or discharge. Prospective data were collected for 20 months after improvement initiatives began from January 2016 to August 2017. All newborns were eligible if admitted to the BCH NICU at <48 hours after birth. The data excluded newborns hospitalized for <48 hours and those whose mothers were ineligible to provide breast milk (ie, illicit substance use or medications not compatible with breastfeeding). The analysis of breast milk use additionally excluded newborns who were ineligible to receive mother‘s milk due to a medical reason such as milk protein allergy and those who were nil per os (NPO) or had died. If a patient was NPO on day of life 7 but then feeding during the 24 hours before transfer or discharge, they were included in the transfer/discharge analysis. The team reviewed medical records for the presence of a documented in-person IBCLC note and for the type of feeding on infant day of life 7 and during the 24 hours before discharge or transfer.

#### Outcome Measures.

1. Percent of mothers who received a lactation consultation.
2. Percent of infants who received any mother’s milk on infant day of life 7.
3. Percent of infants who received any mother’s milk in the 24 hours before discharge or transfer.

#### Process Measure.

Average hospital day of the first lactation consultation (day of admission defined as hospital day 1).

### Statistical Analysis

Statistical process control charts were used to analyze changes in the measures over time and to compare mean values between baseline measurements and after interventions. A significant change was noted if a shift in the mean occurred during the project period. P-charts displayed the main outcome measures (rates), and X-bar S-charts displayed the process measure (average and SD). Chartrunner software was used to create control charts. All other analysis was done using Microsoft Excel.

#### Ethics.

The institutional review board approved this project.

## RESULTS

During the study period, 495 infants were admitted, 78 of which were excluded because their length of stay was <48 hours, leaving 417 infants for analysis (Fig. 1B). Demographic data for infants eligible for maternal lactation consultation were similar in each period pre- and postintervention with no statistically significant difference between the 2 groups (data not shown). The overall median gestational age was 38 weeks with a range of 23 to 42 weeks. Median birth weight was 2945 g with a range of 520 to 4870 g. Fifty-five percent of patients were male. The median length of stay was 6 days with a range of 1 to 102 days. The surgical service admitted 22% of the patients, and medical services admitted 78%. Discharges to home occurred for 19% of patients, and the remaining patients transferred to an inpatient floor or to an outside hospital (see Supplement Digital Content 1, available at http://links.lww.com/PQ9/A60).

**Fig. 2.**
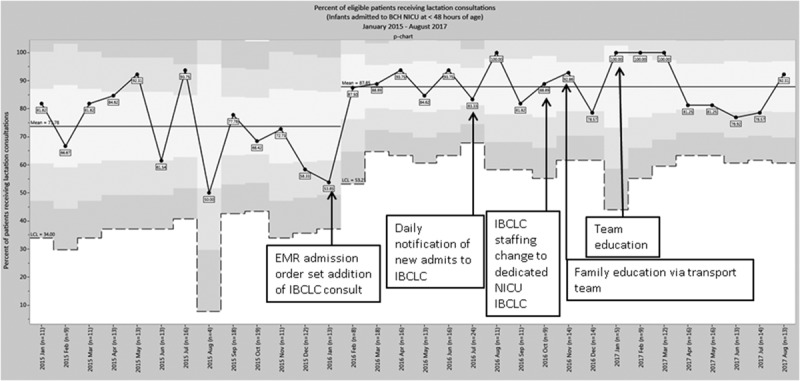
Percent of eligible patients receiving lactation consultations. Control chart analysis after projected initiation showed a significant increase in the percent of eligible patients receiving lactation consults. The results include patients admitted to the BCH NICU at <48 hours after birth and exclude patients admitted for <48 hours and with maternal ineligibility to provide breast milk. The p-chart displays percent of eligible patients receiving lactation consultations over time from January 2015 to August 2017 for infants admitted to the BCH NICU at <48 hours after birth. A significant shift in the mean is noted after project initiation from 73.8% to 87.9%. This change is sustained over time.

The results show that interventions led to an increased rate of lactation consultation, increased breast milk use on day of life 7, and decreased time to first lactation consultation. The percent of eligible patients receiving lactation consults significantly increased from 73% to 87.9% after project implementation (Fig. 2). The statistical process control p-chart shows a significant shift in the mean after project initiation (Fig. 2). This change occurred after the preintervention period of January 2015 to January 2016 (n = 163 patients) compared with the postintervention period of January 2016 to August 2017 (n = 252 patients) and was sustained over time.

**Fig. 3.**
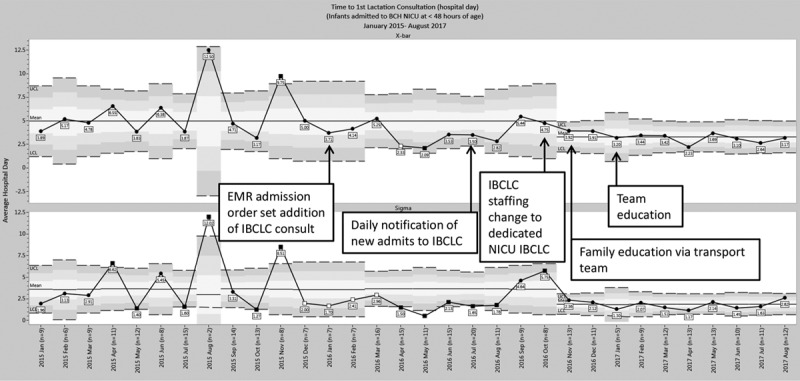
Time to first lactation consultation. Data demonstrate a decrease in time to first lactation consultation during the project period. Eligible patients included patients admitted to the BCH NICU at <48 hours after birth who received lactation consults. Excluded patients were patients who were admitted for <48 hours and maternal ineligibility to provide breast milk. Hospital day 1 was defined as the day of admission. The X-bar chart displays average hospital day at the time of first lactation consultation over time from January 2015 to August 2017 for infants admitted to the BCH NICU at <48 hours after birth. The results show a significant decrease in time to first lactation consultation of 1.7 days from 5 to 3.3 days during the project period, after Plan-Do-Study-Act (PDSA) 3. The Sigma chart with a mean shift from 3.7 to 1.9 describes decreased variability.

Process measure data showed the time to first lactation consultation decreased after project initiation (Fig. 3). The mean shifted from an average of hospital day 5.0 to 3.3 on the X-bar chart (with a day of admission defined as hospital day 1). The sigma chart demonstrated decreased variability (Fig. 3). This decrease occurred after staffing systems changes implemented between January 2015 to October 2016 (n = 226 patients) and November 2016 to August 2017 (n = 109).

**Fig. 4.**
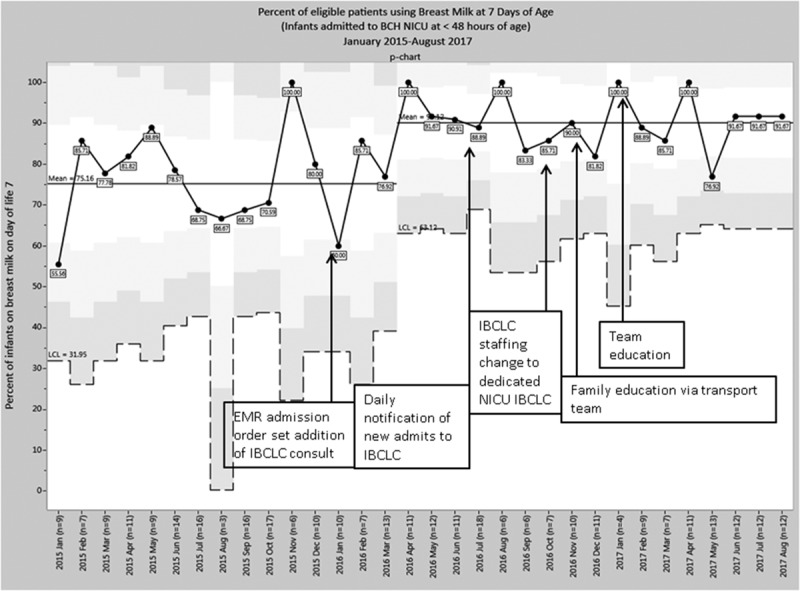
Breast milk use in eligible patients at 7 days of age. Breast milk use in eligible patients on the day of life 7 increased during the project period. Eligible patients included patients admitted to the BCH NICU at <48 hours after birth. Exclusions included patients admitted for <48 hours, deaths, maternal ineligibility to provide breast milk, infants NPO on day of life 7, and medical ineligibility of infant to receive breast milk. P-chart displays the percent of eligible patients receiving breast milk on the day of life 7 over time and displays a shift in the mean from 75.16% to 90.12% after project initiation.

Mean rates of breast milk use on day of life 7 increased from 75.16% to 90.12% after project initiation with a significant shift in the mean on the p-chart (Fig. 4) seen after the first intervention. However, the mean rate of breast milk use at discharge/transfer remained stable with a mean of 90.9% (see Supplemental Digital Content 2, available at http://links.lww.com/PQ9/A61). We excluded patients who were not enterally feeding on day of life 7 from the day of life 7 breast milk use analysis. For these formerly NPO infants who then started enterally feeding during the hospitalization, their rates of breast milk use at the time of discharge or transfer increased from 80% (12/15) preintervention to 88.6% (31/35) postintervention (data not shown). Twenty-nine percent of infants who were not enterally feeding on day of life 7 remained NPO at time of transfer or discharge.

## DISCUSSION

Implementation of an automated electronic lactation consult order, a new method for LC notification, and a consistent IBCLC assignment to the NICU resulted in increased access to lactation services for mothers of infants admitted at <48 hours after birth. The results demonstrated a significantly increased rate of lactation consultations and a decrease in time to the first consultation. The finding of a significantly increased rate of breast milk use on the day of life 7 may reflect the effect of improved access to early lactation support.

Access to lactation services requires prompt notification of the need for consultation and coordinated timing that is medically appropriate for both baby and mother. In a complex, outborn, referral NICU, strategies used at inborn hospital centers would not be relevant, such as prenatal education on arrival to labor and delivery, early pumping in the delivery room, and prompt education at the time of birth. In outborn centers, mothers are often not present at the time of admission. Implementation of an automatic order for lactation consultation into the admission order sets and institution of an automatic notification to the lactation team of new admissions each morning were helpful. Together, these 2 interventions enabled the lactation team to learn of mothers in need of urgent consults rapidly. They then could contact the nurse and mother to determine the best timing of consultation. This coordination was often a critical component of arranging a successful lactation visit. The rate of lactation consultations in infants admitted at <48 hours after birth to the NICU increased to >85%. Both nurses and IBCLCs responded very positively to these interventions. System and personnel changes led to increased support from staff and a sense of ownership of patients and families from the dedicated IBCLC. These system changes could be easily adaptable to other similar NICU settings and have high generalizability in hospitals with electronic ordering systems.

Not only did the rate of lactation consultations increase, but the time to first lactation consultation decreased by 1.7 days. This decrease was important because early lactation consultations play a critical role in supporting early milk expression by mothers.^8^ Improvements in early lactation consultation followed the third Plan-Do-Study-Act cycle that changed to a dedicated, consistent NICU LC. The familiarity of staff with the NICU IBCLC promoted more effective coordination of visits between lactation, nurses, and families. Notably, while time to first lactation consultation decreased, the decrease was only to a mean of hospital day 3.3 (with a day of admission defined as hospital day 1). The lack of in-person access to mothers who were inpatient at outside hospitals can largely explain this finding. However, another barrier is the lack of universal availability of lactation services with only 6 days a week daytime coverage, leaving a gap for 1 weekend day and all nights. To help educate mothers as early as possible, the transport team provided breast milk education sheets to mothers who were inpatient at outside hospitals. The project did not measure the effectiveness of this intervention. A small sample size (n = 4) of qualitative one-on-one interviews with mothers revealed that several mothers did not recall receiving the handouts, but 1 reported it was helpful. The transport team members queried did report any added transport time related to the delivery of the handout. Further work to evaluate the potential value of expanded outreach including balancing measures of possible added system burden is an important next step.

The rate of mother’s milk use on day of life 7 for infants admitted at <48 hours after birth increased during the project period. This work suggests that increased access to lactation services contributed to the increased early breast milk use. The project interventions focused on early initiation and did not address additional challenges that can occur during the sustainment period of lactation. The rate of mother’s breast milk use at the time of discharge or transfer did not show a significant change with the interventions. Rates of breast milk use at the time of transfer or discharge in this population while unchanged were high overall. In general, the BCH NICU often transfers relatively stable newborns to community hospitals, other NICUs, or inpatient medical/surgical units early in their hospitalization period. Due to shorter NICU lengths of stay for many infants compared with other NICUs, the project data may not have captured drop-off of breast milk use later in hospitalization reflective of challenges with the continuation of breastfeeding. The breast milk use outcome measures reported also did not include infants who were not being enterally fed. This formerly NPO subset of infants showed an increased rate of breast milk use at the time of transfer or discharge comparing the baseline to the postintervention period and supported the outcome data of the larger group.

This study had several limitations. The variation in lactation support at maternity centers before mothers’ arrival to the BCH NICU was not measured. The project did not query mothers if lactation services had seen them before the BCH NICU initial consultation. These questions could be investigated in future studies to consider a combined approach to breast milk initiation education between inborn and outborn centers among shared patients. The specific components of the lactation consultation that were most beneficial to influence breastfeeding were not measured, nor did the team explore whether early phone consultation would be a helpful adjunct when mothers are unavailable for an in-person consultation. Only 19% of the newborns in the study transitioned to home after their hospitalization. The remaining newborns transferred to the inpatient floor or to outside hospitals which may limit generalizability. The median gestational age of the population was 38 weeks reflecting the outborn, surgical referral center population. This age may be older than other NICU populations and lead to higher breast milk use rates than achievable at time of discharge in centers with preterm infants with longer lengths of stay where sustainment of breast milk use is challenging. The study focused on early access to LCs but did not address the importance of continued lactation support for mothers after discharge from the NICU whether to another hospital setting or home.

## CONCLUSIONS

Lactation consultation is a critical, time-sensitive component of NICU care. Implementation of quality improvement initiatives to expedite notification of new patient arrivals and to assign a dedicated IBCLC to the NICU led to a substantial increase in the rate of lactation consultations. The time to the first consult significantly decreased, and breast milk use on the day of life 7 increased. These interventions are feasible and generalizable to similar referral units caring for newborns.

## DISCLOSURE

The authors have no financial interest to declare in relation to the content of this article.

## Supplementary Material
