# Supplementary material for: Improving Access to Lactation Consultation and Early Breast Milk Use in an Outborn NICU
Source: Pediatr Qual Saf. 2019 Jan 4;4(1):e130. doi: 10.1097/pq9.0000000000000130 (PMC6426487; doi:10.1097/pq9.0000000000000130)
Supplement: Supplementary file 1 [file pqs-4-e130-s001.docx]

Supplemental Digital Content 1:

Table 1: Patient Characteristics

| **Patient Characteristics (n=417)** | **Result** |
| --- | --- |
| Gestational age at birth  median week (range) | 38 weeks (23-42) |
| Birth weight  median grams (range) | 2945 grams (520-4870) |
| Length of stay  median day (range) | 6 days (1-102) |
| Male sex  N (%) | 218 (55%) |
| Surgical diagnosis  N (%) | 90 (22%) |
| Discharged to home  N (%) | 74 (19%) |
