# Supplementary figures and images for: Improving Access to Lactation Consultation and Early Breast Milk Use in an Outborn NICU
Source: Pediatr Qual Saf. 2019 Jan 4;4(1):e130. doi: 10.1097/pq9.0000000000000130 (PMC6426487; doi:10.1097/pq9.0000000000000130)

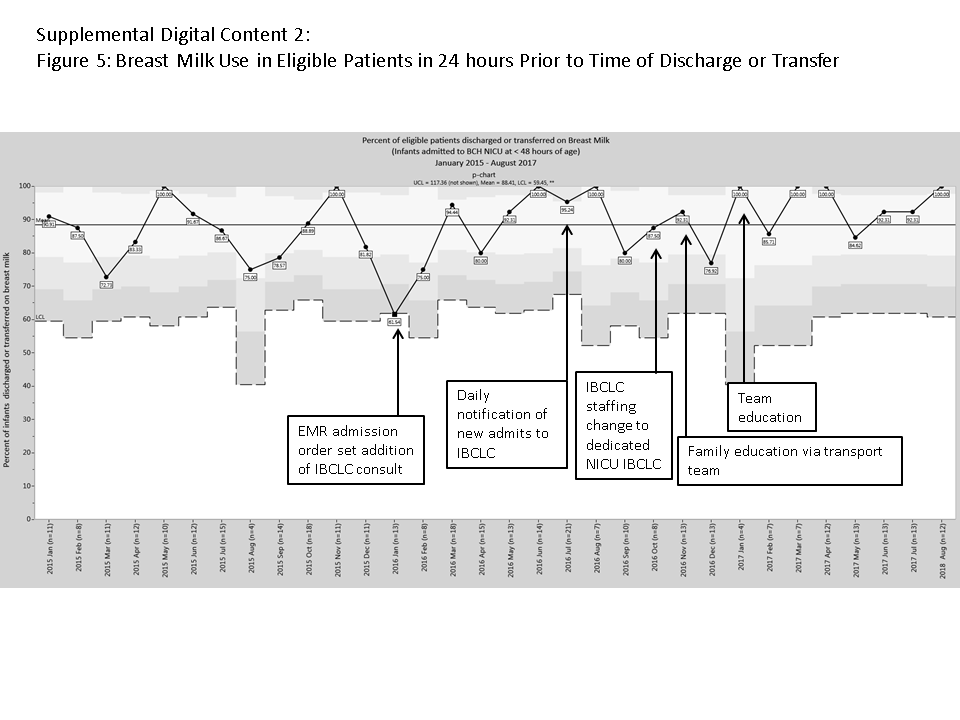

Supplement: Supplementary file 2 [file pqs-4-e130-s002.tif]
